# Association between Tissue Characteristics of Coronary Plaque and Distal Embolization after Coronary Intervention in Acute Coronary Syndrome Patients: Insights from a Meta-Analysis of Virtual Histology-Intravascular Ultrasound Studies

**DOI:** 10.1371/journal.pone.0106583

**Published:** 2014-11-06

**Authors:** Song Ding, Longwei Xu, Fan Yang, Lingcong Kong, Yichao Zhao, Lingchen Gao, Wei Wang, Rende Xu, Heng Ge, Meng Jiang, Jun Pu, Ben He

**Affiliations:** From Department of Cardiology, Ren Ji Hospital, School of Medicine, Shanghai Jiao Tong University, Shanghai, China; S.G.Battista Hospital, Italy

## Abstract

**Background and Objectives:**

The predictive value of plaque characteristics assessed by virtual histology-intravascular ultrasound (VH-IVUS) including fibrous tissue (FT), fibrofatty (FF), necrotic core (NC) and dense calcium (DC) in identifying distal embolization after percutaneous coronary intervention (PCI) is still controversial. We performed a systematic review and meta-analysis to summarize the association of pre-PCI plaque composition and post-PCI distal embolization in acute coronary syndrome patients.

**Methods:**

Studies were identified in PubMed, OVID, EMBASE, the Cochrane Library, the Current Controlled Trials Register, reviews, and reference lists of relevant articles. A meta-analysis using both fixed and random effects models with assessment of study heterogeneity and publication bias was performed.

**Results:**

Of the 388 articles screened, 10 studies with a total of 872 subjects (199 with distal embolization and 673 with normal flow) met the eligibility of our study. Compared with normal flow groups, significant higher absolute volume of NC [weighted mean differences (WMD): 5.79 mm^3^, 95% CI: 3.02 to 8.55 mm^3^; p<0.001] and DC (WMD: 2.55 mm^3^, 95% CI: 0.22 to 4.88 mm^3^; p = 0.03) were found in acute coronary syndrome patients with distal embolization. Further subgroup analysis demonstrated that the predictive value of tissue characteristics in determining distal embolization was correlated to clinical scenario of the patients, definition of distal embolization, and whether the percutaneous aspiration thrombectomy was applied.

**Conclusion:**

Our study that pooled current evidence showed that plaque components were closely related to the distal embolization after PCI, especially the absolute volume of NC and DC, supporting further studies with larger sample size and high-methodological quality.

## Introduction

### Rationale

Distal embolization (DE) is a common complication after percutaneous coronary intervention (PCI), particularly in the setting of acute coronary syndrome (ACS) or vein graft intervention, which may result in microvascular obstruction and no-reflow phenomenon [1], [2]. This undesirable side effect of PCI has been confirmed to be associated with increased post-procedural myocardial infarction, in-hospital mortality, and long-term adverse events [3]–[5]. However, there is no effective strategy for prediction and prevention of DE, which is an important issue for interventional cardiology.

Although several studies using grayscale intravascular ultrasound (IVUS) have indicated that plaque characteristics identified by pre-interventional IVUS (i.e., a large plaque burden, a lipid-pool-like image, and positive remodeling) maybe associated with the angiographic no-reflow phenomenon in ACS patients [6]–[10], gray-scale IVUS is dependent on the simple interpretation of acoustic reflections and of limited value for identifying specific plaque components [11]. Recently, some new methods able to assess both plaque morphology and tissue characteristics, such as virtual histology-IVUS (VH-IVUS), have become clinically available. VH-IVUS is based on spectral and amplitude analysis of IVUS backscattered radiofrequency that allows for characterization of in-vivo atherosclerotic plaque into four types: fibrous (FT), fibrofatty (FF), necrotic core (NC), and dense calcium (DC) [12]–[23]. However, whether pre-PCI plaque characteristics of culprit lesion assessed by VH-IVUS could predict post-PCI angiographic DE, and which plaque components are associated with no-reflow phenomenon remain debated. We therefore performed a systematic review that pooled current evidence to investigate the relationship between pre-PCI plaque composition characteristics assessed by VH-IVUS and post-PCI DE phenomenon in ACS patients.

## Methods

### Search strategy

PubMed, Ovid, EMBASE, and the Cochrane Library databases were searched in their entirety from January 2002 to April 2013. Complex search strategies were formulated using the following MESH terms and text words: intravascular ultrasound, virtual histology, IVUS, VH-IVUS, plaque component, plaque composition, plaque characteristic, no reflow, DE, microembolization, and obstruction. In order to identify any studies missed by the literature searches, we had searched reference lists of all eligible studies and relevant review articles. In addition, we searched from published and ongoing trials in clinical trial registries (ClinicalTrials.gov, Controlled-trials.com and the WHO International Clinical Trials Registry Platform). Searches were not restricted by language, time published, or publication status. Duplicate reports were eliminated (Appendix S1).

### Study selection

We included studies when the following criteria were met:(1) Plaque characteristics were assessed by VH-IVUS; (2) VH-IVUS was performed before coronary intervention in ACS patients; and (3) DE was defined according to angiographic evidence or clinical relevancy. Studies without normal flow (NF) group were excluded from our analysis.

### Data extraction

Two reviewers (D. S. and P. J.) assessed the eligibility of studies using a standardized form developed for this purpose in duplicate and independently. Disagreements were adjudicated by resolved by consensus. Data extraction was completed by the same observers using a standardized data extraction form developed for this study. The following information was extracted from each study: sample size, mean age, gender distribution, risk factors, clinical scenario, definition of DE, and the volume (mm^3^)and percentage of each tissue component of plaque (including FT, FF, NC, and DC). Several studies met our inclusion criteria but were missing data vital to our analysis; in these cases, we contacted the authors to obtain raw data whenever possible.

### Statistical analysis

Statistical analysis in this study was carried out using RevMan software version 5.2 (The Cochrane Collaboration). Results were summarized as weighted mean differences (WMD) with their associated 95% confidence intervals (CI) using both fixed and random effects models, the latter was more conservative where heterogeneity beyond that expected by chance alone was encountered. In addition, the odds ratio (OR) was calculated for baseline comorbidities. Heterogeneity between studies was analyzed by the Q statistic and the I^2^ statistic. A *p* value of the Q statistic <0.1 was defined as an indicator of heterogeneity, and an I^2^ <50% indicated that the magnitude of the heterogeneity might not be significant. Funnel plots were plotted to investigate possible small study effects/publication bias by using Revman 5.2. Planned subgroup analyses were conducted based on the clinical scenario, definition of DE, and whether percutaneous aspiration thrombectomy was applied.

### Quality assessment

Methodological quality was assessed independently by 2 reviewers (D. S. and P. J.) using the Newcastle-Ottawa Scale.

## Results

### Search result

**Figure 1:**
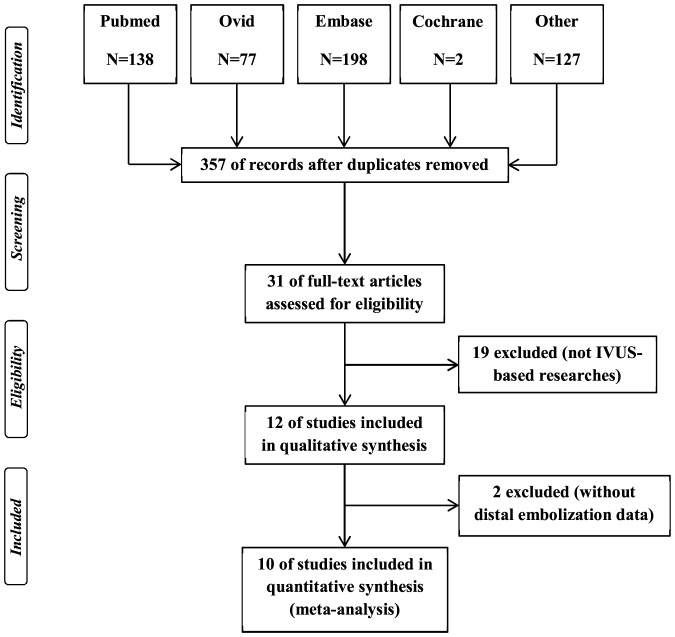
Process of study selection.

After initial literature search, we identified 388 potential studies, of which 357 studies were excluded based on the title and abstracts, because they were unrelated papers, reviews, editorials, letters, case reports or animal studies. The remaining 31 articles were considered of interest and examined in full-text. Of these, 19 studies those were not IVUS-based were excluded. Of the remainder, 2 studies without DE data were excluded [12], [13]. Therefore, 10 observational studies were included in our final meta-analysis [14]–[23]. Figure 1 shows the study selection process.

### Characteristics of included studies

**Figure 2:**
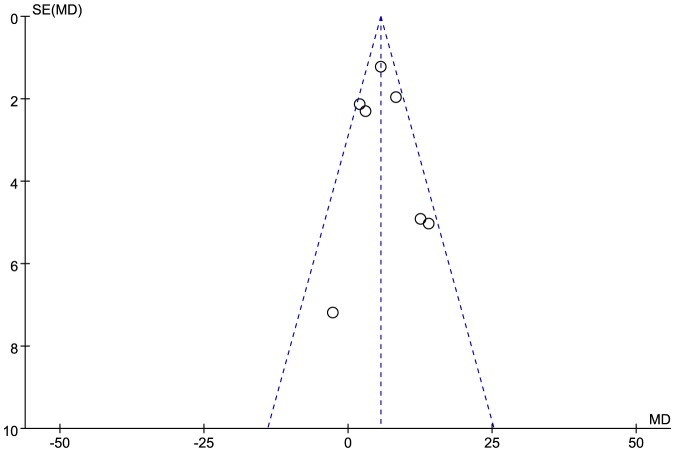
Funnel plot for necrotic core volume outcome data of involved studies.

Table 1 and 2 summarize the main features of the included studies. A total of 872 patients (199 patients in DE group and 673 patients in NF group) were enrolled in the 10 studies, and the sample sizes were 44–190 in each study. Among the included studies, 5 studies involved AMI patients [14], [18]–[21] (4 of them only involved STEMI patients), 3 studies enrolled unstable angina (UA) patients [16], [22], [23], and the remaining 2 studies involved ACS (including both AMI and UA) patients [15], [17]. Percutaneous aspiration thrombectomy was performed before IVUS examinations in 7 studies [14], [15], [18]–[21], [23]. There were no significant differences between DE and NF groups in age and gender of patients. Moreover, there was no significant difference in the incidence of hypertension (OR: 1.36, 95% CI: 0.95 to 1.95, *p* = 0.10), diabetes (OR: 1.36, 95% CI: 0.94 to 1.96, *p* = 0.10) and hyperlipidaemia (OR: 1.44, 95% CI: 0.89 to 2.31, *p* = 0.13) between the two groups. A Funnel plot for NC volume outcome data was used to assess any potential small study effects or publication bias (Figure 2). The Funnel plot was roughly symmetrical as to the mean-effect size line.

**Table 1:** Basical characteristics of studies included in meta-analysis (Normal flow vs. distal embolization).

Study	Study interval	Location	Sample size (n)	Design	Clinical scenario	PAT	Definition of distal embolization
Bae 2008	NR	Daejeon, South Korea	45/12	RSC	AMI	Yes	TIMI flow grade ≤2
Higashikuni 2008	2005.6–2006.4	Tokyo, Japan	40/9	RSC	ACS (AMI and UA)	Yes	Decrease of at least 1 grade in TIMI
Hong 2009	NR	Washington DC, United States	42/38	RSC	UA	No	cTnI elevation >3X the ULN
Hong 2011	2006.2–2008.1	Gwangju, South Korea	166/24	RSC	ACS (STEMI, NSTEMI and UA)	No	TIMI flow grade ≤2
Kawaguchi 2007	2005.8–2006.12	Gunma, Japan	60/11	PSC	AMI (STEMI)	Yes	ST-segment re-elevation
Nakamura 2007	2006.1–2006.3	Saitama, Japan	42/8	PSC	AMI (STEMI)	Yes	Decrease in TIMI flow grade
Ohshima 2009	2007.1–2007.12	Ehime, Japan	24/20	PSC	AMI (STEMI)	Yes	TIMI flow grade ≤2
Ohshima 2011	NR	Ehime, Japan	19/34	RSC	AMI (STEMI)	Yes	TIMI flow grade ≤2
Shin 2011	NR	Ulsan, South Korea	90/22	RSC	UA	No	CK-MB elevation >1X the ULN
Zhao 2013	2010.9–2011.11	Zhengzhou, China	145/21	RSC	UA	Yes	TIMI flow grade ≤2

ACS, acute coronary injury; AMI, acute myocardial infarction; NR, not reported; NSTEMI, non ST-segment elevation myocardial infarction; PAT, percutaneous aspiration thrombectomy; PSC, prospective single center; RSC, retrospective single center; STEMI, ST-segment elevation myocardial infarction; ULN, upper limit of normal.

**Table 2:** Clinical characteristics of studies included in meta-analysis (Normal flow vs. distal embolization).

Study	Mean age (years)	Males (%)	Comorbidities			Pre-PCI use of Aspirin (%)	Use of Statins	Use of GP IIb/IIIa inhibitor	Use of Statins	Use of distal protectiondevices
			HT (%)	DM (%)	HL (%)					
Bae 2008	56.2/67.5	82.2/66.7	40.0/33.3	13.3/33.3	31.1/25.0	100/100	NR	0/0	NR	No
Higashikuni 2008	66.6/60.6	92.5/77.8	70.0/55.6	30.0/55.6	65.0/8	30.0/44.4	22.5/22.2	0/0	22.5/22.2	No
Hong 2009	65/63	47.6/76.3	64.3/73.7	23.8/31.6	NR	92.9/86.8	NR	14.3/10.5	NR	No
Hong 2011	60.5/60.1	65.7/58.3	52.4/70.8	19.3/25	NR	NR	NR	23.5/29.2	NR	NR
Kawaguchi 2007	NR	NR	68.3/81.8	41.7/9.1	45.0/81.8	NR	NR	0/0	NR	No
Nakamura 2007	65.3/58.5	85.7/87.5	42.9/25.0	23.8/37.5	61.9/62.5	100/100	NR	0/0	NR	No
Ohshima 2009	66.0/74.0	83.3/65.0	58.3/75.0	54.2/60.0	62.5/45.0	100/100	NR	NR	NR	No
Ohshima 2011	73/67	68.4/85.3	78.9/70.6	26.3/38.2	47.4/61.8	100/100	NR	NR	NR	No
Shin 2011	61.4/65.5	61.1/50.0	51.1/72.7	32.2/22.7	53.3/63.6	100/100	21.1/27.3	0/0	21.1/27.3	No
Zhao 2013	51/49	66.2/66.6	59.3/61.9	26.9/47.6	NR	100/100	98.6/95.2	NR	98.6/95.2	No

DM, diabetes mellitus; HT, hypertension; HL, hyperlipidaemia; NR, not reported;

Moreover, we evaluated the quality of primary studies using the Newcastle–Ottawa Scale, a validated technique for assessing the quality of observational and non randomized studies. As shown in Table 3, all observational studies were intermediate to low intermediate bias risk as assessed by the Newcastle-Ottawa Scale for quality assessment risk evaluation of adequacy of selection, comparability of study groups, and assessment of outcome or exposure.

**Table 3:** Newcastle-Ottawa Scale of bias risk for the involved studies.

Study	Adequacy of selection			Comparability	Outcomes assessment		
	Representativeness of the exposed cohort	Selection of the non-exposed cohort	Ascertainment of exposure		Assessment of Outcomes	Follow-up period long enough for outcome to occur	Adequacy of follow-up period among cohorts
Bae 2008	**	**	***	***	**	**	**
Higashikuni 2008	**	**	***	***	***	**	**
Hong 2009	**	**	**	***	**	**	**
Hong 2011	**	**	***	***	**	**	**
Kawaguchi 2007	**	**	**	**	**	**	**
Nakamura 2007	**	**	***	***	***	**	**
Ohshima 2009	**	**	***	***	**	**	**
Ohshima 2011	**	**	***	***	***	**	**
Shin 2011	**	**	**	**	***	**	**
Zhao 2013	**	**	***	**	**	**	**

Asterisks are the star rating as per the Newcastle-Ottawa Scale; ** and *** indicate highest rating for these categories.

### Relationship between coronary plaque characteristics and DE

**Figure 3:**
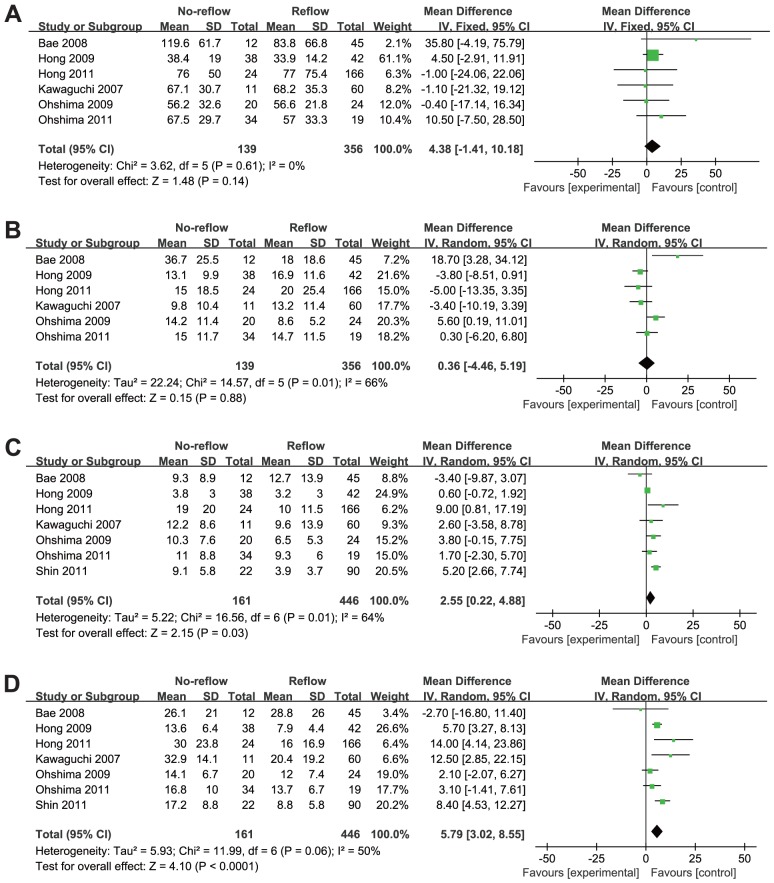
Absolute volume comparison of four different plaque compositions through the entire culprit lesion between the normal flow group and the distal embolization group. (A) Absolute fibrous volume comparison; (B) Absolute fibrofatty volume comparison; (C) Absolute dense calcium volume comparison; (D) Absolute necrotic core volume comparison;

**Figure 4:**
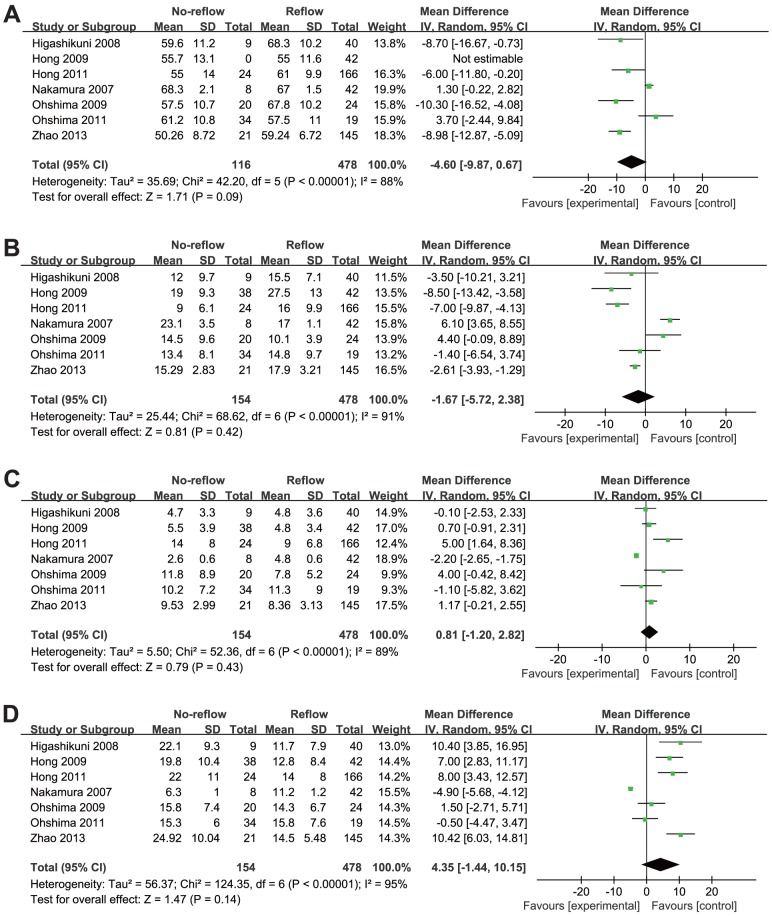
Percentage comparison of four different plaque compositions through the entire culprit lesion between the normal flow group and the distal embolization group. (A) Fibrous percentage comparison; (B) Fibrofatty percentage comparison; (C) Dense calcium percentage comparison; (D) Necrotic core percentage comparison.

As shown in Table 4, Figure 3 and 4, the absolute volume and percentage of four different plaque compositions through the entire culprit lesion were assessed. Compared with NF group, the overall pooled results with random-effects analysis showed DE group had significant higher absolute volume of NC (WMD: 5.79 mm^3^, 95% CI: 3.02 to 8.55 mm^3^; *p*<0.001) and DC (WMD: 2.55 mm^3^, 95% CI: 0.22 to 4.88 mm^3^; *p* = 0.03). The difference between the two groups was not statistically significant with respect to percentage of NC (WMD: 4.35%, 95% CI: −1.44% to 10.15%; *p* = 0.14) and DC (WMD: 0.81%, 95% CI: −1.20% to 2.82%; *p* = 0.43). In addition, there were no significant differences in absolute volume and percentage of FT and FF at the entire culprit lesions between the two groups. Substantial statistical heterogeneity was detected in all of the comparisons among these trials, except for the absolute volume of FT (I^2^  = 0%).

**Table 4:** Composition of plaque by VH-IVUS.

Study		Absolute volume (mm^3^)				Percentage (%)			
		FT	FF	DC	NC	FT	FF	DC	NC
Bae 2008	Reflow	83.8 (66.8)	18.0 (18.6)	12.7 (13.9)	28.8 (26.0)	NR	NR	NR	NR
	No reflow	119.6 (61.7)	36.7 (25.5)	9.3 (8.9)	26.1 (21.0)	NR	NR	NR	NR
Higashikuni 2008	Reflow	NR	NR	NR	NR	68.3 (10.2)	15.5 (7.1)	4.8 (3.6)	11.7 (7.9)
	No reflow	NR	NR	NR	NR	59.6 (11.2)	12.0 (9.7)	4.7 (3.3)	22.1 (9.3)
Hong 2009	Reflow	33.9(14.2)	16.9(11.6)	3.2(3.0)	7.9(4.4)	55.0(11.6)	27.5(13.0)	4.8(3.4)	12.8(8.4)
	No reflow	38.4(19.0)	13.1(9.9)	3.8(3.0)	13.6(6.4)	55.7(13.1)	19.0(9.3)	5.5(3.9)	19.8(10.4)
Hong 2011	Reflow	77 (75.4)	20 (25.4)	10 (11.5)	16 (16.9)	61 (9.9)	16 (9.9)	9 (6.8)	14 (8.0)
	No reflow	76 (50.0)	15 (18.5)	19 (20.0)	30 (23.8)	55 (14.0)	9 (6.1)	14 (8.0)	22 (11.0)
Kawaguchi 2007	Reflow	68.2 (35.3)	13.2 (11.4)	9.6 (13.9)	20.4 (19.2)	NR	NR	NR	NR
	No reflow	67.1 (30.7)	9.8 (10.4)	12.2 (8.6)	32.9 (14.1)	NR	NR	NR	NR
Nakamura 2007	Reflow	NR	NR	NR	NR	67.0 (1.5)	17.0 (1.1)	4.8 (0.6)	11.2 (1.2)
	No reflow	NR	NR	NR	NR	68.3 (2.1)	23.1 (3.5)	2.6 (0.6)	6.3 (1.0)
Ohshima 2009	Reflow	56.6 (21.8)	8.6 (5.2)	6.5 (5.3)	12.0 (7.4)	67.8 (10.2)	10.1 (3.9)	7.8 (5.2)	14.3 (6.7)
	No reflow	56.2 (32.6)	14.2 (11.4)	10.3 (7.6)	14.1 (6.7)	57.5 (10.7)	14.5 (9.6)	11.8 (8.9)	15.8 (7.4)
Ohshima 2011	Reflow	57.0 (33.3)	14.7 (11.5)	9.3 (6.0)	13.7 (6.7)	57.5 (11.0)	14.8 (9.7)	11.3 (9.0)	15.8 (7.6)
	No reflow	67.5 (29.7)	15.0 (11.7)	11.0 (8.8)	16.8 (10.0)	61.2 (10.8)	13.4 (8.1)	10.2 (7.2)	15.3 (6.0)
Shin 2011	Reflow	NR	NR	3.9 (3.7)	8.8 (5.8)	NR	NR	NR	NR
	No reflow	NR	NR	9.1 (5.8)	17.2 (8.8)	NR	NR	NR	NR
Zhao 2013	Reflow	NR	NR	NR	NR	59.24 (6.72)	17.90 (3.21)	8.36 (3.13)	14.50 (5.48)
	No reflow	NR	NR	NR	NR	50.26 (8.72)	15.29 (2.83)	9.53 (2.99)	24.92 (10.04)

Data are presented as mean (SD)

FT, fibrous tissue; FF, fibrofatty; NC, necrotic core; DC, dense calcium.

### Subgroup analysis

Planned subgroup analyses were conducted based on the different clinical scenario, definition of DE, and whether percutaneous aspiration thrombectomy was applied (Table 5 and 6). Subgroup analysis by different clinical scenario showed that patients with DE had significantly higher absolute volume and percentage of NC (WMD: 6.61 mm^3^, 95% CI: 4.11 to 9.12 mm^3^; p<0.001 and WMD: 8.64%, 95% CI: 5.29% to 11.99%; p<0.001) in subgroup of UA patients.

**Table 5:** Subgroup analyses of the association of the absolute volume of plaque components with the onset of distal embolization.

	FT		FF		DC		NC	
Subgroup	WMD (95% CI, mm^3^)	*p*	WMD (95% CI, mm^3^)	*p*	WMD (95% CI, mm^3^)	*p*	WMD (95% CI, mm^3^)	*p*
**Clinical scenario**								
AMI	5.21 (−4.93, 15.35)	0.31	3.17 (−3.25, 9.59)	0.33	1.81 (−0.81, 4.44)	0.18	3.56 (−0.37, 7.50)	0.08
Unstable angina	4.50 (−2.91, 11.91)	0.23	−3.80 (−8.51, 0.91)	0.11	2.77 (−1.73, 7.27)	0.23	6.61 (4.11, 9.12)	<0.001
**Definition of distal embolization**								
Angiographic evidence	5.62 (−4.83, 16.07)	0.29	3.11 (−3.77, 10.00)	0.38	2.44 (−1.34, 6.22)	0.21	3.88 (−0.62, 8.38)	0.09
Clinical relevancy	3.84 (−3.12, 10.80)	0.28	−3.67 (−7.54, 0.20)	0.06	2.71 (−0.88, 6.29)	0.14	7.13 (4.40, 9.87)	<0.001
**Thrombectomy**								
With thrombectomy	−4.36 (−10.42, 1.71)	0.16	0.80 (−3.89, 5.49)	0.74	0.06 (−2.17, 2.30)	0.96	3.56 (−0.37, 7.50)	0.08
Without thrombectomy	−2.57 (−9.14, 3.99)	0.44	−7.38 (−9.86, −4.90)	<0.001	2.59 (−1.60, 6.77)	0.23	7.47 (4.25, 10.69)	<0.001

FT, fibrous tissue; FF, fibrofatty; NC, necrotic core; DC, dense calcium; WMD, weighted mean differences; AMI, acute myocardial infarction.

**Table 6:** Subgroup analyses of the association of the percentage of plaque components with the onset of distal embolization.

	FT		FF		DC		NC	
Subgroup	WMD (95% CI, %)	*p*	WMD (95% CI, %)	*p*	WMD (95% CI, %)	*p*	WMD (95% CI, %)	*p*
**Clinical scenario**								
AMI	−1.48 (−8.40, 5.44)	0.68	3.49 (−0.68, 7.66)	0.10	−0.18 (−3.89, 3.53)	0.92	−1.67 (−6.04, 2.70)	0.45
Unstable angina	−4.34 (−13.81, 5.14)	0.37	−5.06 (−10.75, 0.63)	0.08	0.97 (−0.07, 2.02)	0.07	8.64 (5.29, 11.99)	<0.001
**Definition of distal embolization**								
Angiographic evidence	−4.60 (−9.87, 0.67)	0.09	−0.61 (−4.91, 3.70)	0.78	0.87 (−1.49, 3.24)	0.47	3.91 (−2.30, 10.11)	0.22
Clinical relevancy	0.70 (−4.75, 6.15)	0.80	−8.50 (−13.42, −3.58)	<0.001	0.70 (−0.91, 2.31)	0.39	7.00 (2.83,11.17)	0.001
**Thrombectomy**								
With thrombectomy	5.21 (−4.93, 15.35)	0.31	3.17 (−3.25, 9.59)	0.33	1.81 (−0.81, 4.44)	0.18	3.07 (−3.45,9.59)	0.36
Without thrombectomy	3.98 (−3.07, 11.04)	0.27	−4.09 (−8.19, 0.01)	0.05	3.85 (−0.46, 8.17)	0.08	7.45 (4.38, 10.53)	<0.001

FT, fibrous tissue; FF, fibrofatty; NC, necrotic core; DC, dense calcium; WMD, weighted mean differences; AMI, acute myocardial infarction.

In order to assess the impact of the definition of DE in determining DE on our analyses, subgroup analysis by angiographic or clinical relevance definition was performed. The results showed that there was significantly higher absolute volume of NC (WMD: 7.13 mm3, 95% CI: 4.40 to 9.87 mm3; p = 0.04) in subgroup of DE in clinical relevance definition.

In order to investigate whether percutaneous aspiration thrombectomy would affect the outcomes, trials were divided into two subgroups according to whether thrombectomy was applied. The results showed that in the subgroup without thrombectomy, patients with DE had significantly higher absolute volume and percentage of NC (WMD: 7.47 mm3, 95% CI: 4.25 to 10.69 mm3; p<0.001 and WMD: −7.45%, 95% CI: 4.38% to 10.53%; p<0.001), and significantly lower absolute volume of FF (WMD: −7.38 mm3, 95% CI: −9.86 to −4.90 mm3; p<0.001).

## Discussion

The present meta-analysis that pooled all currently available published data indicated that, among four phenotypes of coronary plaque composition assessed by VH-IVUS, absolute volume of NC components was closely related to the DE after PCI in ACS patients. Besides, absolute volume of DC component might also be related to the DE after PCI. Further subgroup analysis revealed that the predictive value of VH-IVUS plaque characteristics in determining DE was correlated to the clinical scenario of the patients, the definition of DE, and whether percutaneous aspiration thrombectomy was applied.

Two recent review/meta-analyses [24], [25] that investigated the relationship between plaque characteristics and DE after PCI have also reported that the extent of NC was larger in patients with DE. The meta-analysis by Jang et al. [24] evaluated the effect of plaque characteristics on embolization after PCI by grayscale-IVUS and VH-IVUS, and found that the morphologic characteristics of plaque derived from grayscale-IVUS (i.e.,eccentric plaque, ruptured plaque, and attenuated plaque) and the NC component derived from VH-IVUS are closely related to the DE phenomenon after PCI. The systematic review by Claessen et al. [25] summarized the published data on the use of plaque composition assessment by VH-IVUS to predict the occurrence of DE, and found that the NC component was associated with DE in all but 2 of the 11 reviewed studies. In the present study, we performed a systematic review that pooled all the currently available published data investigating the relationship between pre-PCI plaque composition characteristics assessed by VH-IVUS and post-PCI DE phenomenon in ACS patients, and we updated the meta-analysis by adding two VH-IVUS studies [22], [23] that did not include in the previous meta-analysis by Jang et al. [24]. We found that absolute volume of NC component, but not percentage of NC component, was closely related to the DE after PCI in ACS patients, confirming the findings of previous review/meta-analyses. In addition, our analysis that pooled all current evidence found that besides NC volume, absolute DC volume was also closely related to the DE phenomenon after PCI. There is some evidence which indicated that DC might be related to DE. For example, pathologic studies revealed that coronary calcification is related to the total plaque burden, NC component, plaque erosion or rupture that is responsible for coronary thrombosis [26]–[29]. In addition, some studies have also reported that coronary calcium was associated with coronary event including myocardial infarction or death in symptomatic/asymptomatic persons [30]–[33].

In our analysis, we noted a considerable degree of heterogeneity among the included trials. Thus, we performed further subgroup analyses and tried to appraise the possible sources of differences and heterogeneity among trials. Our results suggested that the clinical scenario of the patients, the definition of DE and the use of thrombectomy may influence the correlation between tissue characteristics of coronary plaque and DE. When analyzed in the context of clinical scenario, increased absolute volume of NC was found in DE group in studies including UA patients, but not in those including AMI patients. This phenomenon might be explained by the rupture and migration of NC plaque in AMI patients. In addition, VH-IVUS is limited to detecting thrombus (in fact, thrombus appears as either fibrotic or fibrofatty plaque depending on the age of the thrombus) [34]. Moreover, large amount of NC may have migrated into the distal coronary bed before or during primary PCI in AMI patients [14]. An unexpected finding in our analysis was that no association was found between plaque components and DE in the subgroup of angiographically defined DE. Although angiography has been commonly used as a gold standard for assessing DE, TIMI flow grade is a subjective method to assess epicardial blood flow. As suggested by published literatures, the qualitative nature of TIMI grade renders it somewhat dependent on the technical skill of the observer, and significant differences were found in inter-observer variabilities among different reports, particularly for TIMI 2 grade (Kappa value was 0.4963 for inter-observer variability), which may introduce selection bias in the enrollment of the participants [35], [36].

In addition, our results indicated that pre-stent percutaneous aspiration thrombectomy may also be a important factor in determining the predictive value of VH-IVUS-derived plaque characteristics.

### Study limitations

Several important limitations of our study should be taken into account, in order to place our findings in the proper context. Firstly, as mentioned above, there was considerable heterogeneity in patient characteristics, use of pre-stent thrombectomy, and definitions of DE among the included trials. Secondly, although the consensus document recommends measurement of the absolute and relative components of each plaque at the minimum lumen site and over the whole lesion, these measurements were not usually reported uniformly in the individual studies involved in our study. Thirdly, although our pooled analysis found that besides NC volume, absolute DC volume was also closely related to the DE phenomenon after PCI, more evidence should be obtained to confirm this finding because only two of the studies included in our meta-analysis reported statistically significant association between DC component and post-PCI DE. Finally, all of the involved trials were non-randomized studies and of small sample sizes, which might have brought some bias. Therefore further studies with larger sample size and high-methodological quality are needed.

## Supporting Information

Appendix S1 **Search strategy.**
(DOC)

Checklist S1 **Meta-Analysis on Genetic Association Studies Checklist.**
(DOCX)
