# Supplementary material for: Association between Tissue Characteristics of Coronary Plaque and Distal Embolization after Coronary Intervention in Acute Coronary Syndrome Patients: Insights from a Meta-Analysis of Virtual Histology-Intravascular Ultrasound Studies
Source: PLoS One. 2014 Nov 6;9(11):e106583. doi: 10.1371/journal.pone.0106583 (PMC4222782; doi:10.1371/journal.pone.0106583)
Supplement: Appendix S1 — Search strategy. (DOC) [file pone.0106583.s001.doc]

**1. " Ultrasonography, Interventional "[Mesh] (13998)**

**2. intravascular ultrasound (7114)**

**3. virtual histology (4735)**

**4. IVUS (2941)**

**5. VH-IVUS (164)**

**6. plaque component (2037)**

**7. plaque composition (2724)**

**8. plaque characteristic (1478)**

**9. 1 or 2 or 3 or 4 or 5 or 6 or 7 or 8 (27509)**

**10. "No-Reflow Phenomenon"[Mesh] (223)**

**11. no reflow (2775)**

**12. distal embolization (2758)**

**13. micro-embolization (27)**

**14. micro-obstruction (1)**

**15. 10 or 11 or 12 or 13 or 14 (5412)**

**16. 9 and 15 (138)**
