# Supplementary material for: Association between Tissue Characteristics of Coronary Plaque and Distal Embolization after Coronary Intervention in Acute Coronary Syndrome Patients: Insights from a Meta-Analysis of Virtual Histology-Intravascular Ultrasound Studies
Source: PLoS One. 2014 Nov 6;9(11):e106583. doi: 10.1371/journal.pone.0106583 (PMC4222782; doi:10.1371/journal.pone.0106583)
Supplement: Checklist S1 — Meta-Analysis on Genetic Association Studies Checklist. (DOCX) [file pone.0106583.s002.docx]

**Meta-analysis on Genetic Association Studies Checklist | PLOS ONE**

|  | Item | Section name and paragraph number within manuscript |
| --- | --- | --- |
|  | **Introduction** |  |
| 1 | Provide a detailed justification for the polymorphism studied; if a single polymorphism was analyzed, give details as to why others were not included in the meta-analysis. | N/A |
| 2 | Provide a detailed justification for the population(s) and clinical condition studied. | Introduction (Rationale).  Paragraph 1 |
|  | **Methods** |  |
| 3 | Provide full details of the search strategy employed; outline the full electronic search strategy –specific combination of keywords and any limits applied- for at least one database. Specify whether synonyms of polymorphisms/genes (e.g. SNP number) were searched. | Methods(Search strategy)  Paragraph 3 |
| 4 | Report full details on the inclusion and exclusion criteria applied for selecting studies.  *Please list the excluded articles and the reasons for exclusion of each article in a supplementary file.* | Methods(Study selection, Data extraction)  Paragraph 4 and 5 |
| 5 | Provide details on how the quality of the studies included in the analyses was assessed. | Methods(Quality Assessment)  Paragraph 7 |
| 6 | Describe steps taken to contact study authors to identify additional studies and to request missing data. | Methods( Data extraction)  Paragraph 5 |
| 7 | Describe how environmental effects were adjusted for, if this adjustment was not conducted, outline the reasons for this. | Not conducted. It is not a meta-analysis of polymorphism. |
| 8 | Describe the methods of handling heterogeneity/between-study variance. | Methods( Statistical analysis)  Paragraph 6 |
| 9 | Describe how the Hardy-Weinberg equilibrium and linkage disequilibrium were assessed. | N/A |
| 10 | Describe and justify the choice of model for the analyses (per-allele vs per-genotype vs genetic model-free, random effects vs fixed effects). | Methods( Statistical analysis)  Paragraph 6 |
| 11 | Describe whether a sensitivity analysis has been completed. | N/A |
| 12 | Describe whether an assessment of the effects of population stratification has been conducted. | N/A |
| 13 | Describe whether study-specific results have been assessed and if so the reasons for this (e.g. forest plot). | N/A |
|  | **Results** |  |
| 14 | Include flow diagram for the studies included in the meta-analysis as the first figure for the manuscript | Results(Search result)  Paragraph 8 |
| 15 | Report details on allele/genotype prevalence. | N/A |
| 16 | Report the effect size estimates and p values for each analysis. | Results(Relationship between coronary plaque characteristics and DE,Subgroup analysis)  Paragraph 11-14 |
|  | **Discussion** |  |
| 17 | Discuss the limitations of the meta-analysis, including genotyping errors/bias and publication bias. | Study limitations  Paragraph 18 |
| 18 | If the meta-analysis identifies an association within a subgroup of the population studied but not another, discuss the implications of these results, and if applicable the possibility of subgroup-specific publication bias. | Discussion  Paragraph 17 |
| 19 | Discuss the suitability of the sample size employed to the research question and the power of the study. | Study limitations  Paragraph 18 |
